# Ultrasound-Assisted Extraction of Total Acetogenins from the Soursop Fruit by Response Surface Methodology

**DOI:** 10.3390/molecules25051139

**Published:** 2020-03-03

**Authors:** Gabriela Aguilar-Hernández, María de los Ángeles Vivar-Vera, María de Lourdes García-Magaña, Napoleón González-Silva, Alejandro Pérez-Larios, Efigenia Montalvo-González

**Affiliations:** 1 Laboratorio Integral de Investigación en Alimentos, Tecnológico Nacional de México-Instituto Tecnológico de Tepic. Av. Tecnológico 2595 Fracc. Lagos del Country, Tepic, Nayarit 63175, Mexico; gaby.mca2017@gmail.com (G.A.-H.); mgarciam@ittepic.edu.mx (M.d.L.G.-M.); 2 Tecnológico Nacional de México/Campus-Instituto Tecnológico de Tuxtepec. Depto. de Ingeniería Química y Bioquímica-Maestría en Ciencias en Alimentos. Tuxtepec, Oaxaca 68350, Mexico; angelesvivar@hotmail.com; 3 División de Ciencias Agropecuarias e Ingenierías, Centro Universitario de los Altos, Universidad de Guadalajara, Av. Rafael Casillas Aceves 1200, Tepatitlán de Morelos, Jalisco 47600, Mexico; napoleon.gonzalez@cualtos.udg.mx (N.G.-S.); alarios@cualtos.udg.mx (A.P.-L.)

**Keywords:** *Annona muricata*, soursop fruit, acetogenins, ultrasound-assisted extraction, response surface methodology

## Abstract

The soursop fruit or *Annona muricata* (*A. muricata)* fruit is recognized by its bioactive compounds and acetogenins (ACG) are among the most important. The effect of ACGs, with greater importance in health, is that they present anti-tumor activity; however, the methods of extraction of ACGs are very slow and with a high expenditure of solvents. To our knowledge, there is no report of an optimal method for the extraction of acetogenins from the Annonaceae family by ultrasound-assisted extraction (UAE); therefore, the aim was to find the best UEA conditions of acetogenins from *A. muricata* fruit (peel, pulp, seed, and columella) by using response surface methodology. The effect of amplitude (40%, 70%, and 100%), time (5, 10, and 15 min), and pulse-cycle (0.4, 0.7, and 1 s) of ultrasound at 24 kHz was evaluated on the total acetogenin content (TAC). Optimal extraction conditions of acetogenins (ACGs) with UEA were compared with the extraction of ACGs by maceration. The optimal UEA conditions in the *A. muricata* pulp and by-products were dependent on each raw material. The highest TAC was found in the seed (13.01 mg/g dry weight (DW)), followed by the peel (1.69 mg/g DW), the pulp (1.67 mg/g DW), and columella (1.52 mg/g DW). The experimental TAC correlated well with the model (Adjusted *R*^2^ with values between 0.88 and 0.97). The highest effectiveness in ACG extraction was obtained in seeds and peels using UEA compared to extraction by maceration (993% and 650%, respectively). The results showed that *A. muricata* by-products are an important source of ACGs and that UAE could be a viable alternative, with high potential for large-scale extraction.

## 1. Introduction

The *Annona muricata* tree grows in tropical and subtropical areas and its fruit is commonly known as the soursop fruit or graviola. *Annona muricata* is one of the main cultivated species of the Annonaceae genus and is widely distributed in Mexico, Brazil, Colombia, Venezuela, Cuba, and India [1]. The fruit (peel, pulp, and seeds) and parts of the tree (stems, roots, and leaves) were the subject of numerous researches, due to their content of bioactive compounds, with the acetogenins (ACGs) among the most important [2,3,4].

ACGs are molecules with an aliphatic chain of 35 to 37 carbon atoms, attached to one, two, or three rings of tetrahydrofuran or tetrahydropyran in their central region. The chain also has several oxygenated groups and a terminal α-β-unsaturated or satured γ-lactone, sometimes is rearranged as ketolactone [5,6]. Low-dose acetogenins have important biological activity; they can inhibit the mitochondrial Nicotinamide adenine dinucleotide (NADH) ubiquinone oxidase reductase (Complex I of the respiratory chain), which reduces the production of ATP [7,8]. Several studies reported that *A. muricata* extracts (mainly from leaves and seeds) have an anti-tumor effect on several cell lines of breast, prostate, liver, and lung cancer among others, with the anti-tumor activity attributed to acetogenins [9,10,11].

Currently, the raw extract of acetogenins from *Annonaceae* is obtained principally by maceration, percolation, or solid–liquid extraction [5,12]. However, these techniques require the use of large volumes of solvents, application of heating, and long extraction times. In this sense, it is necessary to look for alternative extraction methods that help to reduce the amount of solvent, extraction times, and losses of the compounds of interest.

Ultrasound (US) produces a phenomenon known as cavitation which contributes to the rupture of the cellular wall, the reduction of the pore size of solid materials, and an increase in the contact surface area between the solid phase and the solvent, facilitating the extraction of the components [13,14]. This technology was widely used for a great diversity of compounds [3,15,16], but not for the extraction of acetogenins. In a single study reported by Leon-Fernandez et al. [17], they found that the use of ultrasonic bath at 42 kHz for 3 h increased the qualitative extraction of acetogenins from soursop pulp compared to extraction by maceration, soxhlet method, and microwaves; however, further trials are required using an ultrasonic equipment with direct application, evaluating factors of amplitude, extraction time, temperature, and ultrasonic cycles (pulse-cycles), to optimize the extraction in different raw materials and different bioactive compounds [18].

Response surface methodology (RSM) is an effective mathematical and statistical tool, which is widely used to evaluate multiple parameters and their possible interactions between variables in various experimental processes. RSM is useful to develop, improve, and optimize processes in which a response of interest is influenced by several variables and the goal is to optimize the response [19,20].

The objective was to extract ACGs from *A. muricata* peel, pulp, seed, and columella, using ultrasound-assisted extraction (UAE) to optimize the extraction conditions (extraction time, sonication amplitude, and pulse-cycles) by using RSM. Additionally, the results obtained with the UAE under optimal conditions of total ACGs extraction were compared with a conventional extraction method.

## 2. Results and Discussion

### 2.1. Effect of Ultrasound-Assisted Extraction (UAE) on the Total Acetogenin (TAC) Content from A. Muricata Pulp and By-Products

The results obtained for the TAC are presented in Table 1. Significant statistical differences of the TAC between treatments can be observed (*p* < 0.05). The TAC from *A. muricata* peel, pulp, seeds, and columella depended on the experimental conditions to which they were subjected and the structural composition of the plant matrix used. According to Table 1, the highest TAC was found from the seeds (13.01 mg/g dry weight (DW)), followed by the peel (1.55 mg/g DW) and pulp (1.67 mg/g DW) under the same conditions of amplitude (X_SA_ 100%) and pulses (X_PC_ 0.7 s), with extraction times of 15 min for seed and peel, and 5 min for pulp. Meanwhile, the highest TAC from columella (1.52 mg/g DW) was obtained with 10 min of extraction, 40% amplitude, and 0.4 s of pulse-cycle.

The effect of UAE on the extraction of ACGs is discussed as follows: the application of ultrasonic waves had mechanical and thermal effects producing cavitation in the solvent; the microbubbles interacted with the vegetal cells and modify their structure by breaking down the cell walls and/or increasing the size of cell pores, resulting in a faster dissolution of the bioactive compounds in the solvent [21,22,23]. On the other hand, the differences of the UAE conditions for extracting ACGs also depend on the structural chemical composition of each raw material and the physical–mechanical phenomenon generated by each UAE combination. The higher amplitude or ultrasonic density, the longer treatment time, and the constant acoustic irradiation cause high cavitation, which in turn leads to the most significant cell lysis [21]. Thus, the peel and seeds that have a higher content of cellulose, hemicellulose, and lignin [24] required longer cavitation to extract the ACGs as opposed to the pulp and columella.

Champy et al. [7] extracted ACGs from *Annona muricata* pulp by using maceration and they found 0.52 mg annonacin/kg DW. Gromek et al. [25] reported 1% of annonacin from *A. muricata* seeds by using liquid–solid extraction; however, the authors did not divulge the total ACG content. On the other hand, Yang et al. [26] found 1.67–2.29 mg/g from seeds of different *Annona* species by using supercritical fluid extraction. León-Fernández et al. [17] reported the qualitative presence of acetogenins in soursop pulp using soxhlet, microwave, and ultrasonic bath extraction (3 h, 42 kHz). This experiment demonstrated that ultrasound is an efficient method of extraction.

The TAC from each raw material also varied significantly, and this can be attributed to the fact that each one synthesizes ACGs in a different proportion. ACGs are biosynthesized in roots, stems, leaves, peel, and fruit pulp, and their concentration depends on the type of tissue in which they are found and their different stages of development [27]. Although the role of acetogenins for the plant is not entirely known, their toxicity against arthropods or insects suggests that they constitute forms of chemical defense for all parts of the plant [4,27]. It was demonstrated that ACGs are biosynthesized during the organogenesis of the seeds, and it is suggested that ACGs can be used as antimicrobials or toxic compounds for animals, implying an early defense mechanism against phytopathogens or predators as a survival strategy in this species [28].

### 2.2. Analysis of the Response Surface Methodology

**Figure 1:**
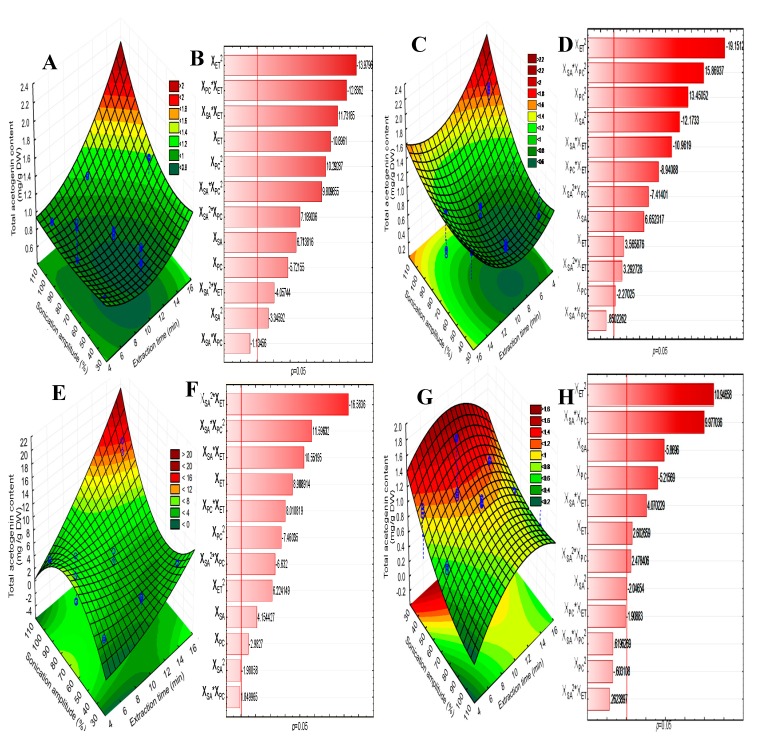
Response surface plots and Pareto charts of the total acetogenin content from *A. muricata* peel (**A**, **B**), pulp (**C**, **D**), seed (**E**, **F**), and columella (**G**, **H**) after the ultrasonic-assisted extraction. DW, dry weight; X_ET_, extraction time (min); X_SA_, sonication amplitude (%); X_PC_, pulse cycle.

The response surface plots (Figure 1) show the effects of the significant interactions between the different UAE conditions on the TAC from *A. muricata* peel (Figure 1A), pulp (Figure 1C), seed (Figure 1E), and columella (Figure 1G), where the elliptical shape of the plots indicates the interactions between the corresponding variables [29]. UAE extraction of ACGs was performed throughout the experimental domain and in all samples tested, regardless of extraction time, pulse-cycles, and sonication amplitude used. The Pareto charts for each sample (Figure 1B,D,F,H) indicate that the effect (negative or positive) of the variables on the TAC is matrix-dependent and the effect of the independent variables and their interactions in each raw material could be classified as follows: peel, pulp, and seed X_ET_ > X_PC_ > X_SA_ and columella X_ET_ > X_SA_ > X_PC_.

RSM was developed with the TAC data as a function of extraction time, pulse-cycles, and sonication amplitude, using the multiple regression technique. Table 2 shows the second-order polynomial equations for each sample, which describe the individual and combined effect of all independent variables on the TAC. Moreover, the analysis of variance showed that the experimental data from the peel, pulp, seed, and columella have a good correlation (*R*^2^ = 0.97, 0.98, 0.97, and 0.91, respectively) and an adequate adjustment of the experimental data with the model (lack of fit, *p* > 0.05). The lack of fit demonstrated the suitability of the model, indicating an approximation to the real system. According to the RSM, the extraction of acetogenins from *A. muricata* peel, pulp, seed, and columella can be described using a predicted mathematical model.

### 2.3. Assessment of Model Reliability and Comparison of UAE with Maceration Extraction

Table 3 shows the optimal conditions of the UAE for extracting acetogenins from *A. muricata* peel, pulp, seed, and columella. To verify the reliability of the model, the ACG extraction was performed using the UAE optimal conditions for each sample as suggested by Aydar et al. [30]. The results obtained from the TAC (Table 4) for all samples matched with the predicted data under UAE optimal conditions. Table 3 shows that, to obtain the highest TAC from the peel, pulp, and seed, 100% sonication amplitude, mean cycle-pulses (0.7 and 0.55 s), and extraction times of 15 min for peel and seed and 5 min for pulp are required. The maximum TAC from columella was obtained with 10 min extraction, 0.4 s cycle-pulses, and a 40% amplitude.

On the other hand, Table 4 shows the results obtained of the TAC from each sample, using the optimal UAE conditions and comparing the TAC obtained with maceration. The highest TAC was observed in all samples when the UAE was used, obtaining the highest extraction effectiveness at short times (5 and 15 min) compared to the 168 h used with maceration. Furthermore, the TAC was 650%, 407%, 993%, and 271% higher for peel, pulp, seed, and columella, respectively, when UAE was applied compared to maceration.

## 3. Materials and Methods

The experimental development of this research was carried out in two stages. In the first stage, acetogenins were extracted from the *A. muricata* fruit (pulp and by-products), following the conditions established in the Box–Behnken experimental design and, subsequently, the optimal UAE conditions to extract acetogenins were determined, using RSM. In the second stage, the UAE method was validated by extracting the highest acetogenin content under the optimal UAE conditions and the results were compared with those obtained by the conventional method.

### 3.1. Plant Material

The soursop fruits (*Annona muricata* L.) were collected in orchards located in Compostela, Nayarit, Mexico, and were used until they reached consumption maturity (15–19° Brix). Peel, pulp, seeds, and columella were manually separated and freeze-dried (Labconco 700201000, Kansa, MO, USA) at −50 °C and pressure of 0.12 mbar. The samples were ground in a food processor (Nutribullet NB-101B, Los Angeles, CA, USA) and sieved on a No. 35 stainless-steel mesh (Fisher Scientific, Hampton, NH, USA) to a particle size of 500 µm.

### 3.2. Ultrasonic-Assisted Extraction (UAE)

UAE of acetogenins from *A. muricata* pulp and by-products was carried out using a Box–Behnken design with three levels for each factor (see Table 1). Individual and interaction effects of extraction time (X_TE_ 5, 10, and 15 min), pulse-cycle (X_PC_ 0.4, 0.7, and 1 s), and sonication amplitude (X_AS_ 40%, 70%, and 100%) on total acetogenin content (TAC) were evaluated. The experiments were carried out in a randomized manner to reduce unexplained effects of variability in the observed response.

ACG extraction was developed using a UP400S ultrasonic system (400 W, 24 kHz frequency) (Hielscher Ultrasonics, Teltow, Germany). The ultrasonic probe (H7 Tip 7, Hielscher, Teltow, Germany), with a 100% maximum amplitude corresponding to 175 μm and acoustic power density of 300 W/cm^2^, was submerged to 2 cm of the extractant solution. The procedure started with 2 g of each lyophilized sample in an extraction tube with 10 mL of chloroform. The ultrasonic extraction was performed according to the experimental design. A water bath (Thermo Scientific 2870, Waltham, MA, USA) was used to maintain the temperature (25 ± 2 °C) and then the samples were centrifuged (Hermle Z32HK, Wehingen, Germany) at 8000 rpm for 5 min at 4 °C and the supernatants were collected for analysis.

### 3.3. Total Acetogenin Content (TCA)

The content of ACGs was determined by considering the colorimetric reaction of ACGs with Kedde’s reagent; this reaction gives a pink color when Kedde’s reagent reacts with α-β-unsaturated or satured γ-lactone [17,31,32,33]. The acetogenic extracts (50 µL) were mixed with 2 mL of Kedde’s reagent, and the absorbances were measured at 505 nm in a spectrophotometer (Jenway 6705, Dunmow, UK). A standard annonacin curve was performed (*R*^2^ = 0.9745) and the results were expressed in milligram equivalents of annonacin per gram of dry weight (mg/g DW).

### 3.4. Analysis of the Response Surface Methodology (RSM)

RSM was applied to obtain the optimal UAE conditions for each raw material. A second-order polynomial equation derived from the RSM was used to calculate the predicted response (Equation (1)).

(1) $$Y\;=\;\beta 0\;+\;\overset{Ε}{\underset{i\;=\;A}{\sum }}\beta i\;Xi\;+\;\overset{Ε}{\underset{i\;=\;A}{\sum }}\overset{Ε}{\underset{j\;=\;A\;\neq \;i}{\sum }}\beta \mathrm{ij}\;Xi\;+\;Ε,$$

where *Y* is the predicted response (TAC), *X*i is the coded or uncoded value for the factors (*X*_TE_, *X*_PC_, and *X*_AS_), β0 is a constant, βi is the main effect of the coefficient for each variable, and βij represents the interaction effect coefficients. The adequacy of the model was evaluated by analysis of variance (ANOVA) to determine the effects of significant interactions in the model (*p* < 0.05) and by quantification of the coefficient of determination (*R*^2^ and adjusted *R*^2^).

### 3.5. Model Reliability and Comparison of UAE with Conventional Extraction

To verify the reliability of the model, the UAE was again performed on each sample using the optimal extraction conditions provided by the RSM. The results obtained from the TAC under the optimal conditions were compared with the results obtained by the conventional extraction method (maceration). The effectiveness of the UAE respect for conventional extraction was also calculated.

### 3.6. Extraction of Acetogenins by Maceration

The extraction of ACGs by maceration was performed with 2 g of lyophilized sample into 10 mL of chloroform at room temperature for one week. After maceration, the TAC was quantified as described in Section 2.2.

### 3.7. Effectiveness of UAE in the Acetogenin Extraction

The effectiveness of the UAE in the extraction of acetogenins was calculated using Equation (2).

(2) $$\mathrm{Effectiveness}\;\left(\%\right)\;=\;\frac{\mathrm{TAC}\;\mathrm{by}\;\mathrm{UAE}\;}{\mathrm{TAC}\;\mathrm{by}\;\mathrm{maceration}}\;\times \;100\;$$

### 3.8. Statistical Analysis

Data were expressed as means ± standard deviation (*n* = 3). In the first stage, the RSM was used, and, in the second stage, the experimental data were analyzed in a unifactorial design. The analyses were developed using an analysis of variance (ANOVA) (*p* < 0.05) with the Statistica v.10 program (Statsoft, Tulsa, Oklahoma, USA). Least significant difference (LSD) test was used to analyze the difference between means (α = 0.05).

## 4. Conclusions

The results obtained in this research show that it was possible to optimize, through response surface methodology, the best UAE conditions to extract acetogenins with short extraction times (5 and 15 min) compared to maceration. The extraction effectiveness increased by 2–10-fold depending on the raw material. The present study also highlights that *A. muricata* seed and peel could be a potential source of acetogenic compounds. This research is the first study where UAE was used to increase the acetogenin extraction from soursop fruit; however, this study can also form the basis for the investigation of different extraction solvents, as well as for heat and ultrasound treatments such as thermosonication.

## Figures and Tables

**Table 1:** Effect of the process variables of ultrasound-assisted extraction on total acetogenin content (TAC) from *Annona muricata* peel, seed, columella, and pulp.

Run	Ultrasonic Conditions			Total Acetogenins (mg/g Dry Weight (DW))			
	X_ET_	X_SA_	X_PC_	Peel	Pulp	Seed	Columella
1	5	40	0.7	0.87 ± 0.02 ^gC^	0.73 ± 0.02 ^fgD^	2.84 ± 0.18 ^hiA^	0.95 ± 0.05 ^dB^
2	15	40	0.7	0.81 ± 0.01 ^iC^	1.10 ± 0.01 ^dB^	4.25 ± 0.08 ^dA^	0.83 ± 0.01 ^eC^
3	5	100	0.7	0.95 ± 0.02 ^fC^	1.67 ± 0.05 ^aB^	4.08 ± 0.15 ^deA^	0.49 ± 0.05 ^hD^
4	15	100	0.7	1.55 ± 0.02 ^aB^	1.37 ± 0.03 ^bC^	13.01 ± 0.47 ^aA^	0.79 ± 0.01 ^efD^
5	5	70	0.4	0.89 ± 0.02 ^gB^	0.72 ± 0.01 ^fghC^	9.19 ± 0.77 ^bA^	0.63 ± 0.06 ^gD^
6	15	70	0.4	1.36 ± 0.01 ^bB^	1.17 ± 0.01 ^cC^	3.15 ± 0.11 ^ghA^	0.84 ± 0.01 ^eD^
7	5	70	1	1.31 ± 0.02 ^cB^	0.72 ± 0.01 ^fgC^	3.04 ± 0.10 ^fghA^	0.65 ± 0.05 ^gD^
8	15	70	1	1.06 ± 0.01 ^eB^	0.63 ± 0.05 ^iD^	2.71 ± 0.13 ^iA^	0.66 ± 0.02 ^gC^
9	10	40	0.4	1.08 ± 0.02 ^eC^	0.72 ± 0.04 ^fgD^	4.18 ± 0.05 ^deA^	1.52 ± 0.01 ^aB^
10	10	100	0.4	1.13 ± 0.01 ^dB^	0.62 ± 0.01 ^iD^	2.68 ± 0.14 ^iA^	0.81 ± 0.05 ^efC^
11	10	40	1	0.90 ± 0.01 ^gB^	0.75 ± 0.04 ^fC^	3.98 ± 0.21 ^deA^	0.76 ± 0.04 ^fC^
12	10	100	1	0.89 ± 0.02 ^gC^	0.70 ± 0.02 ^ghD^	3.80 ± 0.20 ^efA^	1.06 ± 0.04 ^cB^
13	10	70	0.7	0.82 ± 0.02 ^hC^	0.65 ± 0.05 ^iD^	4.06 ± 0.12 ^deA^	1.07 ± 0.02 ^cB^
14	10	70	0.7	0.88 ± 0.01 ^gC^	0.67 ± 0.01 ^hiD^	5.89 ± 0.02 ^cA^	1.14 ± 0.01 ^bB^
15	10	70	0.7	0.67 ± 0.01 ^hD^	0.84 ± 0.02 ^eB^	3.39 ± 0.01 ^fgA^	0.79 ± 0.03 ^efC^

X_ET_, exposition time (min); X_SA_, sonication amplitude (%); X_PC_, pulse cycle. Data are expressed as means ± standard deviation (*n* = 3). Different lowercase letters indicate significant statistical differences between treatments (α = 0.05); different capital letters indicate significant statistical differences between raw materials (α = 0.05).

**Table 2:** The predicted mathematical models for the extraction of acetogenins (mg/g DW) from *A. muricata* peel, seeds, columella, and pulp after ultrasound-assisted extraction.

Product	Polynomial Equation	*R* ^2^	Adjusted *R*^2^
Peel	5.2189 − 0.0692X_SA_ + 0.0002X_SA_^2^ − 10.5455X_PC_ + 6.8201X_PC_^2^ − 0.0577X_ET_ + 0.0082X_ET_^2^ + 0.1684X_SA_ × X_PC_ − 0.0722X_SA_ × X_PC_^2^ − 0.0005X_SA_^2^ × X_PC_ − 0.0014X_SA_ × X_ET_ + 0.0000X_SA_^2^ × X_ET_ − 0.1202X_PC_ × X_ET_	0.97	0.96
Pulp	2.9971 − 0.036X_SA_ − 0.00X_SA_^2^ − 5.8434X_PC_ + 6.5128X_PC_^2^ − 0.1616X_ET_ + 0.0122X_ET_^2^ + 0.0962X_SA_ × X_PC_ − 0.127X_SA_ × X_PC_^2^ + 0.0006X_SA_^2^ × X_PC_ + 0.001X_SA_ × X_ET_ − 0.00X_SA_^2^ × X_ET_ − 0.091X_PC_ × X_ET_	0.98	0.97
Seeds	−8.7493 + 1.3271X_SA_ − 0.0137X_PC_^2^ − 71.5537X_PC_ + 62.9214X_PC_^2^ + 1.7041X_ET_ + 0.0492X_ET_^2^ + 0.5769X_SA_ × X_PC_ − 1.0821X_SA_ × X_PC_^2^ + 0.0070X_SA_^2^ × X_PC_ − 0.1175X_SA_ × X_ET_ + 0.0009X_SA_^2^ × X_ET_ + 0.9514X_PC_ × X_ET_	0.97	0.95
Columella	2.90 − 0.0701X_SA_ + 0.0003X_SA_^2^ − 2.3411X_PC_ – 0.7397X_PC_^2^ + 0.2056X_ET_ − 0.0115X_ET_^2^ + 0.0627X_SA_ × X_PC_ +0.0080X_SA_ × X_PC_^2^ − 0.0003X_SA_^2^ × X_PC_ + 0.0010X_SA_ × X_ET_ − 0.0000X_SA_^2^ × X_ET_ − 0.0321X_PC_ × X_ET_	0.91	0.88

X_SA_, sonication amplitude (%); X_PC_, pulse cycle (s); X_ET_, extraction time (min); R^2^, regression coefficient.

**Table 3:** Optimal conditions of the ultrasonic-assisted extraction to extract acetogenins from *A. muricata* peel, pulp, seed, and columella.

Parameter	Peel	Pulp	Seed	Columella
Extraction time (min)	15	5	15	10
Pulse cycle (s)	0.55	0.7	0.7	0.4
Sonication amplitude (%)	100	100	100	40
Optimal response (mg/g DW)	1.69	1.67	13.01	1.52
−95% Confidence limit	1.63	1.60	12.28	1.42
+95% Confidence limit	1.75	1.73	13.73	1.62
Extraction time (min)	15	5	15	10

−95% confidence limit, lower limit; +95% confidence limit, upper limit; confidence interval, the difference between upper and lower limits; DW, dry weight.

**Table 4:** Total acetogenin content from *A. muricata* peel, pulp, seeds, and columella using the optimal conditions of ultrasonic-assisted extraction (UAE) and its effectiveness in comparison with the maceration extraction (EM).

Parameter/Method	UAE	EM	UAE	EM	UAE	EM	UAE	EM
	Peel		Pulp		Seed		Columella	
Total acetogenins (mg/g DW)	1.69 ± 0.02^b^	0.26 ± 0.01^g^	1.67 ± 0.01^b^	0.41 ± 0.01^f^	13.01 ± 0.02^a^	1.31 ± 0.01^d^	1.52 ± 0.01^c^	0.56 ± 0.02^e^
Effectiveness UAE (%)	650		407		993		271	

Data are expressed as means ± standard deviation (*n* = 3). Different letters (a–f) indicate significant statistical differences between treatments (α = 0.05).
